# Malament stitch and increased risk of bladder neck stenosis: any association following open prostatectomy in Enugu Southeast Nigeria

**DOI:** 10.1186/s12894-021-00944-y

**Published:** 2022-01-13

**Authors:** Okwudili Calistus Amu, Emmanuel Azubuike Affusim, Ugochukwu Uzodimma Nnadozie, Okezie Mbadiwe

**Affiliations:** 1 College of Medicine, University of Nigeria-Enugu Campus, Enugu, Nigeria; 2 College of Medicine, Odumegwu Ojukwu University, Awka, Anambra State Nigeria; 3 Federal University Teaching Hospital, Abakiliki, Ebonyi State Nigeria

**Keywords:** Malament stitch, Open prostatectomy, Bladder neck stenosis

## Abstract

**Background:**

Malament stitch is one of the effective techniques employed to minimize bleeding in simple open prostatectomy but concerns about possibility of increased risk of bladder neck stenosis has limited its routine use.

**Aim:**

We studied patients who had open prostatectomy with Malament stitch to determine the incidence of bladder neck stenosis amongst them.

**Material and methods:**

This was a prospective study of 72patients who had simple open prostatectomy in which malament stitch was applied from 2010 to 2020. A proforma was designed to collect data. Pretreatment variables were transrectal ultrasound (TRUS) volume of prostate, pretreatment IPSS value, postvoidal residual urine volume before surgery, weight of enucleated prostate adenoma, time to removal of Malament stitch. Outcome measures were done with post treatment IPSS and PVR at 6 weeks, 3 months and 6 months. Cystoscopy was done at 3 months or 6 months for patients with rising outcome measures to determine presence of bladder neck stenosis.

**Results:**

The mean age of patients in this study was 68.3 years (SD = 7.1, range 52–82). The mean of the pretreatment score for IPSS was 30.7 (SD = 3.9, range 18–34) and 5.9 (SD = 0.2) for QOLS. The mean weight of prostate estimated with ultrasound was 169.5 g and mean weight of enucleated adenoma of the prostate was 132.5 g. The mean time of removal of Malament stitch was 23.1 h. Only 3 (4.2%) patients required cystoscopy because of increasing IPSS and PVR at 3 months postprostatectomy. 2 (2.8%) patients out of 72patients were confirmed to have bladder neck stenosis at cystoscopy.

**Conclusion:**

Malament stitch did not lead to significant incidence of bladder neck stenosis in this study.

## Introduction

Benign prostatic hyperplasia remains a common disease of aging men [1]. Over the years, improvements in its evaluation and treatment have continued to be witnessed. Presently the gold standard for surgical treatment is transurethral resection of the prostate (TURP) [2]. Laser- based surgeries is also gaining grounds as a possible improvement on TURP [3, 4], however simple open prostatectomy still has its indications in resource poor countries like Nigeria because of the high cost of establishing Laser-based surgical endoscopic suites [5]. Moreover, we are often faced with large prostates [6, 7] and increased likelihood of complications of LUTS like bladder stones, diverticulum that will definitely necessitate an open transvesical prostatectomy [5].

One of the complications of simple open transvesical prostatectomy is bleeding which may be intraoperative or postoperative. Bleeding leads to recurrent episodes of clot retention postoperatively and frequent multiple blood transfusions with its attendant risks. Over the years, one of the haemostatic stitch developed was the Malament stitch which significantly stopped or reduced postoperative bleeding and incidence of clot retention [8–13].

However a lot of concern has arisen over the possibility of increased incidence of bladder neck stenosis as a late complication following the application of Malament stitch [14]. This has limited its use and many surgeons are not keen on acquiring the skill of applying it.

We studied prospectively patients who had transvesical prostatectomy with Malament stitch applied in an attempt to determine if there was an increased risk of bladder neck stenosis in such patients in our own environment.

## Patients and methods

This is a prospective study carried out at 82 Division Military Hospital in Enugu state in Nigeria from 2010 to 2020. An average of 72 prostatectomies are performed yearly in the hospital. Simple open prostatectomies account for an average of 12 while TURP account for the rest in a year. 82patients were recruited into the study. Ethical clearance was obtained from 82 division military hospital health research ethics committee and informed consent obtained from patients to include them in the study. Research was carried out in accordance with relevant guidelines and regulations of the institution’s ethics committee. All patients who had open transvesical prostatectomy in which Malament stitch was applied were recruited into the study and followed up for 1 year. Patients recruited were established to have BPH. Patients who had PSA above 4 ng/ml had prostate biopsies to rule out cancer of the prostate. All patients were optimized. Hypertension and diabetes mellitus were controlled. Patients with deranged clotting profile were excluded from the study. Patients with deranged kidney function were placed on continuous drainage until kidney function normalized. Patients on antiplatelets stopped the drugs for 4 weeks before recruitment into the study. Severity of lower urinary tract symptoms were initially assessed with international prostate symptom score (IPSS) and postvoidal urine volume (PVR) for those who were not on catheter at presentation. Maximum flow rate was discarded because many of the patients presented to outpatients clinic in acute retention, acute on chronic retention or chronic retention and were already on urethral catheter. Patients whose enucleated prostate adenoma revealed cancer on histology were also excluded from the study.

**Fig. 1:**
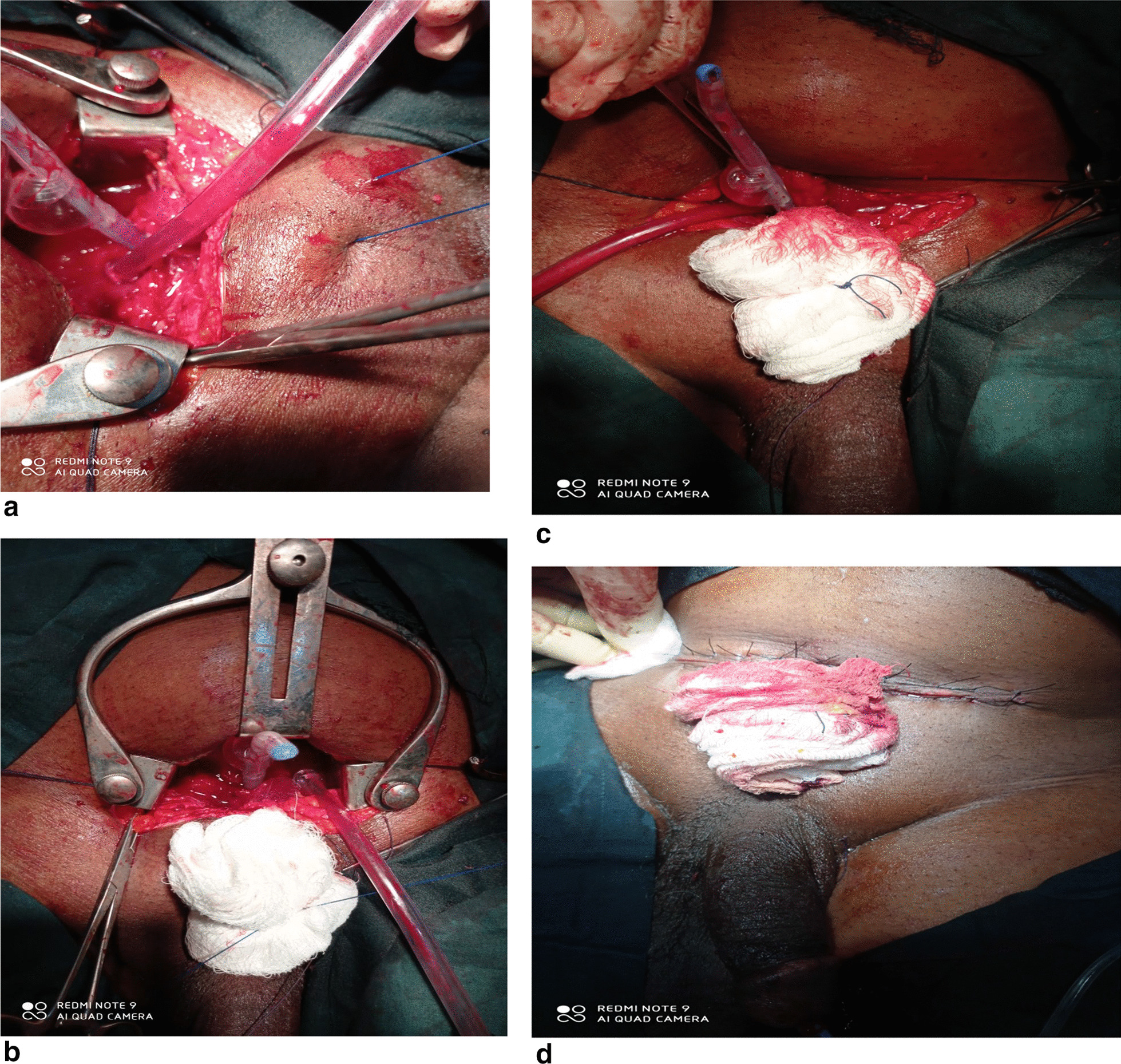
**a** Malament stitch (nylon) in place occluding bladder neck with only catheter passing through. **b** Malament stitch being tied under tension over a roll of gauze separating bladder neck from prostatic fossa and allowing only catheter to pass through. **c** completed malament suture knotted over a roll of gauze. **d** wound closure completed

The procedure: spinal anaesthesia was used for all patients. Patient is placed on supine position, routine cleaning and draping of the lower abdomen was done. A Pfannenstiel incision was done two fingerbreaths above the pubic symphysis. Incision was deepened to the rectus fascia which was incised transversely. Each lip of the incised fascia was developed and a flap of it raised superiorly and inferiorly exposing the rectus muscles and pyrimidalis muscle. The muscles were bluntly separated in the midline exposing the bladder. A Balfour retractor was inserted to keep the rectus muscles separated exposing the bladder more. A gauze on a sponge holding forcep is used to tease off the perivesical fat and mobilize the peritoneal refection superiorly. Bladder is opened longitudinally between two stay sutures making sure that the inferior lip of the incision does not go too close to the bladder neck. Malament and colleagues preferred entering the bladder through a transverse incision but we found a longitudinal incision faster with less bleeding because it avoids the vessels more. Urine is suctioned out. The Balfour retractor is replaced by the Millins retractor which is inserted into the bladder. The internal ureteric orifices are identified. Using diathermy, a semicircular incision is made on the mucosa overlying the median lobe just below the posterior prostatovesical junction. The adenoma is bluntly enucleated carefully avoiding trauma to the prostatic capsule to avoid increased bleeding. Adenoma is removed and the prostatic fossa is immediately packed with hot roll of gauze. The edges of the prostatic capsule with the bladderneck are picked up with Allis tissue forceps. Vicryl 2–0 is used to approximate the mucosa of the bladder neck to the prostatic capsule between the 5′0clock position and 7′0clock position achieving haemostasis. A figure of eight suture is also applied to the 5′0clock and 7′0clock position as described by Harris to further achieve haemostasis using same vicryl 2–0. Malament described placement of the figure of eight suture at 4’O clock and 8’Oclock positions using zero plain catgut however we opted for the more modern Harris modification. A Malament stitch is then applied which involves a non absorbable nylon or prolene2 suture (it is important to note that Malament used No. 2 dermalon non-absorbable suture which was not available) which is introduced about 4 cm below the inferior lip of the skin incision in the midline. It passes through skin, subcutaneous tissue then through the inferior aspect of the rectus fascia flap appearing at the anterior aspect of the bladder neck at 12’O clock position, it traverses the bladder wall into the anterior aspect of the prostatic fossa. The suture is then taken round the bladder neck at its junction with the prostatic capsule making sure to stay closer to the capsule and taking a good bite to avoid avulsion of the tissue. The suture crosses over to the opposite side of the entry point while exiting the bladder and is brought out through the skin at least 3–4 cm opposite its entry point. The gauze pack is then removed and a size 22 haematuric silicone catheter (Malament used size 22 Foleys catheter but we preferred silicon as it is an improvement on Foleys catheter owing to its inert nature)is passed through the urethra into the bladder and its balloon inflated with 10 ml of sterile water (After closure of bladder wall, the sterile water in the balloon is increased to 30 ml. This avoids inadvertent puncture of balloon while closing bladder wall). The Malament suture is then tied over a roll of gauze (Malament described tying over a plastic bridge but we found a roll of gauze readily available) confirming that it is firmly applied to the catheter passing through the bladderneck. In that way the bladder cavity is completely separated from prostatic fossa except for the catheter passing through. Figure 1a–d. The ureteric orifices are observed for spurting of urine to confirm they are not occluded by the Malament stitch. Occasionally furosemide is given to facilitate spurting of urine. The Millins retractor is removed and the bladder is closed in two layers using vicryl 1–0 suture. A retropubic drain is left insitu and the wound closed in layers with nylon 2–0 to skin.

Postoperative management involved removal of Malament stitch between 18 and 36 h post application. Time of removal of malament depended mostly on confirming absence of active soaking of penile dressing by blood. Removal of the Malament stitch is done at the patient’s bedside and causes negligible discomfort so anaesthesia is not required. It involves removing the roll of gauze, cutting one end of the suture at the knotted area and pulling out the suture by exerting gentle traction at the other end. This usually takes less than 2 min.

Urethral catheter was removed between 10 and 14 days. Incidence of clot retention before and after Malament stitch was removed was documented.

Patients lower urinary outcome was assessed at 6 weeks, 3 months and 6 months using IPSS and postvoidal residual urine volume (PVR). To achieve this, the cell phone numbers of the patient and at least one relative was obtained and saved to facilitate invitation of patients.

Patients who had increasing IPSS and or increasing postvoidal urine volume after initial improvement were subjected to cystoscopy after confirming sterile urine on culture within the 1 year follow up period.

Patients with bladder neck stenosis confirmed on cystoscopy were documented and had endoscopic bladder neck incision/resection. A predesigned proforma is used to collect all data.

Results were analyzed using SPSS version 20 with the assistance of a statistician. Results were expressed using tables as means and standard deviation. Pictograms and graphs were used where necessary. Independent sample t test was used to compare means of variables measured to test for significance. P values < 0.05 were considered significant.

## Results

The number of patients recruited for the study was 81. However 9 patients were lost to follow up and could not be traced with their submitted cell phone numbers. They were excluded from the study. 72patients completed the study and were analyzed. The mean age of patients in this study was 68.3 years (SD = 7.1, range 52–82). The mean of the pretreatment score for IPSS was 30.7 (SD = 3.9, range 18–34) and 5.9 (SD = 0.2) for QOLS. The mean weight of prostate estimated with ultrasound was 169.5 g and mean weight of enucleated adenoma of the prostate was 132.5 g. The mean time of removal of Malament stitch was 23.1 h. There was no incidence of clot retention before and after removal of Malament stitch in this study.

Tables 1 and 2 shows a summary of key variables studied.

**Table 1:** Mean and ranges of values of key variables

Variables	All (N = 72)
*Age*	
Mean (SD)	68.3 (7.1)
Median [range]	68 [52–82]
*Weight of patient (kg)*	
Mean (SD)	73.3 (11.0)
Median [Range]	74 [51–97]
*TRUS volume/weight of prostate (g)*	
Mean (SD)	169.5 (76.6)
Median [Range]	153.5 [66–589.5]
*Pretreatment IPSS score*	
Mean (SD)	30.7 (3.9)
Median [range]	32 [18–34]
*Weight of enucleated prostate (g)*	
Mean (SD)	132.5 (59.2)
Median [range]	120 [59–426.9]
*Time of removal of malament stitch postoperatively (in hours)*	
Mean (SD)	23.1 (3.6)
Median [Range]	23.5 [18–23.1]

**Table 2:** PVR responses of patients

Categories of PVR	Percentage	Mean (SD)
On catheter	58.3	–
Not on catheter	41.7	191.0 (183.0)

Only 3 (4.2%) patients required cystoscopy because of increasing IPSS postprostatectomy. 2 (2.8%) patients out of 72patients were confirmed to have bladder neck stenosis at cystoscopy and one patient was found to have partial bulbar stricture.

Comparison of variables in patients who had bladder neck stenosis with those who did not is depicted in Table 3.

**Table 3:** Comparison of variables among the malament stitched patients with or without bladder neck stenosis

Variables	Values across groups		P values
	Group 1: BNS,(n = 2)	Group 2: No BNS (n = 70)	
Weight of patients	75.5	73.24	0.12
TRUS volume/weight of prostate (g)	70.75	172.39	< .00001*
Pre-treatment IPSS Score	25.5	30	0.0072*
Weight of enucleated prostate	59.95	134.41	< .00001*
Time of removal of Malament Stitch post-operatively (Hours)	19.5	23.16	0.4922

^*^Significant with the P < 0.05 for equal variance not assumed

**Fig. 2:**
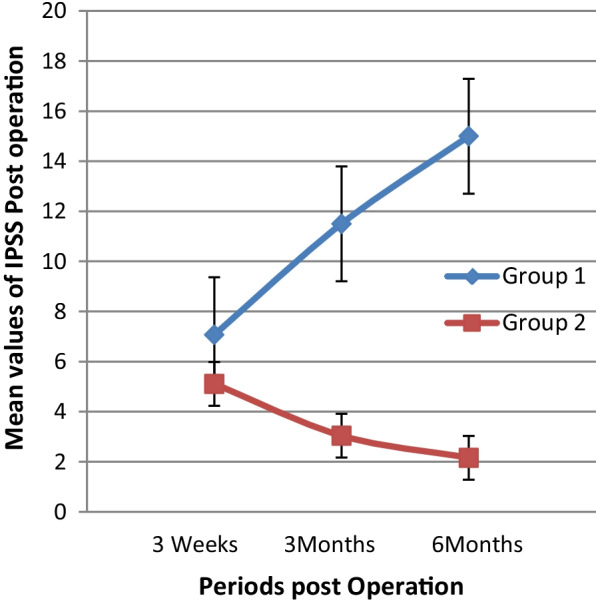
Trends of the Mean values of IPSS post operation

**Fig. 3:**
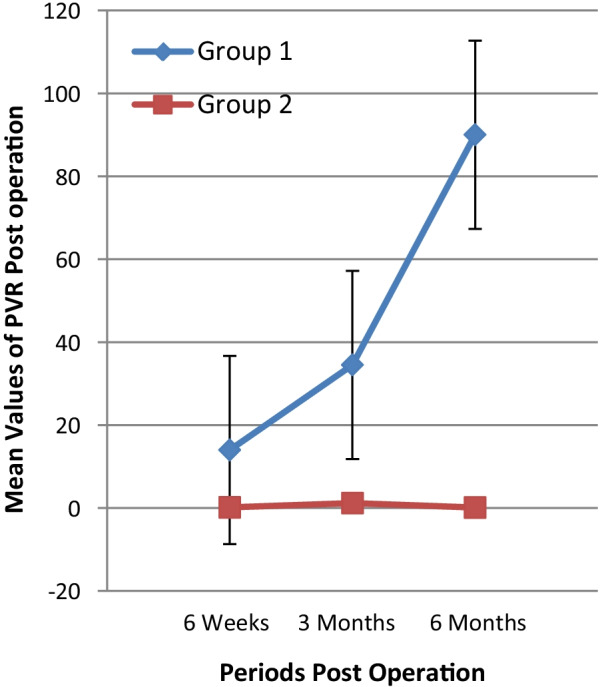
Trends of the Mean values of PVR post operation

The changes in IPSS and PVR in patients with confirmed bladder neck stenosis compared to those without bladder neck stenosis is depicted in Table 4 and in Figs. 2 and 3. The increasing IPSS and PVR necessitated cystoscopy in these patients which finally confirmed bladder neck stenosis.

**Table 4:** Trends of IPSS and PVR post operation

Groups	Periods of follow up post operation		
	6 weeks	3 months	6 months
*IPSS values cross periods post operation*			
Group 1: BNS,(n = 2)	7	11.5	15
Group 2: No BNS (> 6 month follow-up)(n = 70)	5.11	3.04	2.16
*PVR values cross periods post operation*			
Group 1: BNS,(n = 2)	28	34.5	71
Group 2: No BNS (> 6 month follow-up)(n = 70)	0.17	1.20	0.14

## Discussion

Transvesical prostatectomy has remained a veritable tool in the management of BPH. It is invaluable for very big prostates exceeding 100 g, concomitant bladder stones and bladder diverticulum which could be handled at same time and despite the advent of laser-based surgeries for big prostates, it has remained useful. Moreover laser based surgeries is not readily available in resource poor countries. However excessive bleeding and recurrent episodes of clot retentions have been a recurring challenge in transvesical prostatectomy. Several attempts have been made to control bleeding during and after transvesical prostatectomy. Malament et al. [6] came up with this technique that excludes the bladder from the prostatic fossa in an attempt to reduce bleeding using a temporary stitch. Many other researchers have modified the technique with same principle. Malament stitch has been found to reduce blood loss perioperatively in open prostatectomies. Nielsen et al. [11] in a controlled clinical trial of a series of 64 consecutive cases of transvesical prostatectomies followed up for 3 months after operation concluded that the suture significantly reduces perioperative blood loss. Similar results were reported by Nicoll et al. [15] in one of the largest series in which they did a comparative analysis of 300 consecutive cases of removable purse-string sutures. Many other researchers reported similar finding. Morbidity and mortality is linked to blood loss and excessive blood transfusions thus making Malament stitch a veritable tool in transvesical prostatectomies. An often neglected advantage of this suture is in remarkably reducing incidence of clot retention. There was no incidence of clot retention recorded in this study. Clot retention remains a major source of stress for both patient and surgeon necessitating frequent suctioning and flushing of the bladder with tormenting pain that the patient has to endure.

However, the concern has been that the malament stitch may predispose the patient to subsequent bladder neck stenosis as a late complication [14]. We decided to report our own findings in Africans in Enugu State of Nigeria as it is known that Africans because of their pigmentation seem to have exaggerated response to wound healing with likelihood to have increased scarring and formation of hypertrophic scars and keloids [16–19].

Only two patients (2.8%) in this study were found to have cystoscopy confirmed bladder neck stenosis out of 72patients. This figure compares to reported incidence of bladder neck stenosis where malament stitch was not used [20–25]. The reported prevalence of bladder neck stenosis in these studies ranged from 1.7 to 6.3%. Tubaro et al. [25] in one of the few prospective studies on efficacy of suprapubic transvesical prostatectomy in patients with benign prostatic hyperplasia followed up his patients for 1 year and reported 6.25% incidence rate for bladder neck stenosis In other words, this finding was not because Malament stitch was used but may be due to other patient’s factors. Further studies on the patients that had bladder neck stenosis may help elucidate these factors. Dakum et al. [10] in their series of 104 patients found bladder neck contractures in 2 patients who had malament stitch and in one patient amongst those in whom malament stitch was not used. They noted that there was no significant difference statistically between the groups and concluded that malament stitch did not lead to increased incidence of bladder neck stenosis. Nielson [11] after a follow up of 3 months in 64 patients had similar finding. Alfthan et al. [12] also concluded in their study that Malament suture does not increase risk of bladder neck stenosis.

Interestingly, the two patients that had bladder neck contractures had a significant smaller enucleated prostate adenomas compared to those who did not have bladder neck contractures. This may be related to the fibrous nature of smaller obstructing prostates and healing with a more pronounced fibrosis and not necessarily because Malament stitch was applied.

Time of removal of Malament stitch in this study (between 16 and 36 h) had no relationship with development of bladder neck contractures.

It is important to subject a patient to further studies postoperatively once the IPSS and PVR values progressively worsens over time. In this study cystoscopy was done for such patients confirming bladder neck stenosis in two patients and bulbar stricture in one patient. All were successfully treated endoscopically.

### Limitations of the study

Some patients were lost to follow up despite attempt to reach them on submitted cell phone numbers. This was not a randomized controlled study. We did not do a randomized controlled study because this study was carried out in a military hospital and the ethical committee was concerned and uncomfortable that the control arm may bleed more since the malament stitch will not be applied and that may create problems for the hospital. We hope to carry out a randomized controlled study in a teaching hospital in the future. We accept this as a limitation that will hamper drawing significant statistical conclusion.

The duration of follow up was 1 year. It is possible that some BNS may be missed but this is unlikely. Many other quoted studies reported a follow up period of 3 months–1 year.

## Conclusion

Malament stitch applied during transvesical prostatectomy and removed between 16 and 36 h does not increase the possibility of developing bladder neck contracture in our BPH patients. A randomized controlled clinical study will be required to conclude significantly this finding.

### Recommendation

We will encourage urologists to consider applying malament stitch routinely in open transvesical prostectomy to reduce the incidence of bleeding.

## Data Availability

The datasets used and/or analysed during the current study are available from the corresponding author on reasonable request.
